# Obituary

**Published:** 1911-12

**Authors:** R. H. L. Bibb


### OBITUARY.

David R. Wallace, A. M., M. D., LL. D., died at his home in Waco, Texas, on the 21st of November of this year, at the advanced age of 86 years, a half century of which time was spent in the practice of medicine in Texas.

Dr. Wallace was born in Pitt county, North Carolina, November 10, 1825. His paternal grandfather—one of three brothers—came from Scotland to fight against the English during the Revolutionary War, and was with Washington at the surrender of Cornwallis at Yorktown. His boyhood was spent on his father's farm until he was 14 years of age, at which time the family moved to Greenville, N. C., where he entered a primary school and obtained a good preparatory education in English and mathematics; and, although he often made a hand in the field and at the plow, whilst pursuing these studies, such was his assiduity he was able to enter Wake Forrest College and complete its four-year course in three and a half, and to graduate with the first honors of his class, and of which he was the youngest, and of which he was, also, the valedictorian. At the date of his death he was the oldest living graduate of that institution.

After graduating from Wake Forrest, Dr. Wallace, while reading medicine under his preceptor, taught school for several years to obtain funds with which to prosecute his medical studies at college, and was so far successful as to enable him to enter the medical department of the University of New York in the fall of 1853, from which institution he graduated in 1854. During his studies in New York he acted as assistant to Prof. Jno. W. Draper, President of the University, in his demonstrations before his classes. Upon graduating, Dr. Wallace was offered the chair of physiology in the University, and was urged by Professor Draper to accept the appointment. Professor Draper had become deeply attached to, and interested in, his young pupil, an attachment which was mutual and which lasted as long as the professor lived; but, being in very poor health, Dr. Wallace declined the flattering offer, because—as he told the writer—he feared the state of his health would not permit him to discharge the duties of so responsible a position as they should be discharged. He left New York and went to Philadelphia, where he passed the summer in attending a summer course at the University hospital and in assisting the professor of chemistry in his class-room demonstrations. He returned to North Carolina in the fall of 1854, and engaged in a copartnership practice with Dr. E. J. Blount, an old and prominent physician of his home neighborhood; but, as his health continued to fail, in December of that year, fearing he was a victim of tuberculosis, he bade adieu to family, friends and native State, and came to Texas. Soon after coming to Texas, Dr. Wallace was induced to accept, temporarily, the professorship of Latin and Greek in Baylor University, then located at Independence, Texas, where he remained, continued his connection with Baylor University and practiced medicine until that institution was moved to Waco, and the advent of the Civil War.

In 1862, without solicitation, Dr. Wallace was commissioned a surgeon in the Confederate army, and was ordered to duty with the Fifteenth Texas Infantry regiment. He remained in service as regimental and as departmental surgeon until the end of the war, at which time he was surgeon of the department of Southern Texas.

At the close of the war, Dr. Wallace, without a penny in his pockets, located in Waco, formed a copartnership with Dr. J. H. Sears, and enjoyed a large, a successful and a lucrative practice until his appointment to the Superintendency of the State Lunatic Asylum, in 1874, a position he held until 1879, when he went to Waco and resumed the practice of his profession. In 1883 he was appointed Superintendent of the North Texas Lunatic Asylum at Terrell, Texas, and remained in charge of that institution until 1891, when he returned to his old home and devoted the remainder of his professional life to the treatment of nervous diseases.

Dr. Wallace was one of only five honorary members of the American Medical and Psychological Society; he was several times Vice-President of the American Medical Association, one of the founders and the second President of the Texas State Medical Association; President of the McLennan County Medical Society, corresponding member of several European medical bodies, and a member of the American Association of Superintendents of Lunatic Asylums. He was an LL. D. of Baylor University, and whilst not extensive for a man of his culture and ability his contributions to medical literature attracted the attention of leading medical men throughout the world, both for the profoundness of his reasoning and the force and beauty of the language he used.

Dr. Wallace was married to Miss Arabella Daniel, of Independence, in 1857; she died in 1868, and in 1871 he was married to Mrs. Sue Roberts, a widowed sister of his first wife. His widow, two married daughters and several grand children survive him.

Whilst it can be truthfully written that Dr. Wallace adorned every position he occupied during his eventful and brilliant career, unquestionably his master work, that which gave him the greatest satisfaction, was done among the insane of this State. His intimate knowledge of the anatomy, the physiology and the pathology of the human brain, and the many causes that disrupt its normal functions, as well as the methods by which these functions could be restored, coupled with his well recognized administrative ability, has set a mark in asylum management to which none but the best, the most competent, need ever hope to attain.

In the death of Dr. Wallace, his family has lost an affectionate husband, a kind and an indulgent father; the medical profession one of its ablest, most enlightened and successful members; science an untiring student; the State and the community in which he lived a model citizen, whose life was an exemplification of all that is honorable and upright in man, whilst humanity mourns the loss of a noble, open-hearted benefactor.

R. H. L. Bibb, M. D.

